# Supplementary material for: Content-rich biological network constructed by mining PubMed abstracts
Source: BMC Bioinformatics. 2004 Oct 8;5:147. doi: 10.1186/1471-2105-5-147 (PMC528731; doi:10.1186/1471-2105-5-147)
Supplement: Additional File 2 — The original results of the above study (non-essential files are deleted to keep the file size under the limit set by BMC bioinformatics). [file 1471-2105-5-147-S2.bz2 › chilibotAdditionalFile2/dip10/302ID8647433E1293/html/U2AF1_U2AF2.html]

 


 **U2AF1** and **U2AF2** 
  
Found 123 abstracts in PubMed, retrieved 10.  
 

 What does Google say? 
 PDF only 
| .edu only 

---

**Interactive relationship** (e.g. stimulation, inhibition, etc)

**Neutral relationship**- The splicing factor U2AF  [ **U2AF2** ]   [ **U2AF1** ]  65, U2 small nuclear ribonucleoprotein particle snRNP auxillary factor of 65 kDa, binds to pyrimidine rich sequences at 3 splice sites to recruit U2 snRNP to pre mRNAs.  Ref: 12558503 Biochem J, 2003
- Copurification of the recombinant U2AF35  [ **U2AF1** ]  RNA recognition motif U2AF35  [ **U2AF1** ]  RRM and full length U2AF65  [ **U2AF2** ]  yields a soluble and functionally active minimal U2AF  [ **U2AF2** ]   [ **U2AF1** ]  heterodimer.  Ref: 12297299 FEBS Lett, 2002
- Here, we show that the pyrimidine tract of the last splice acceptor site of the human beta globin gene is necessary to stimulate mRNA 3 end formation in vivo and binds the U2AF  [ **U2AF2** ]   [ **U2AF1** ]  65 splicing factor.  Ref: 12189174 EMBO Rep, 2002
- We found that the recombinant U2AF35  [ **U2AF1** ]  RRM is unstructured in solution but its tertiary structure is induced upon binding to U2AF65  [ **U2AF2** ] .  Ref: 12297299 FEBS Lett, 2002
- Sex lethal splicing autoregulation in vivo interactions between SEX LETHAL, the U1 snRNP and U2AF  [ **U2AF2** ]   [ **U2AF1** ]  underlie male exon skipping.  Ref: 12490553 Development, 2003

**Non-interactive relationship** (e.g. studied together, co-existance, homology, etc.)

- The arginine and serine rich domain of U2AF  [ **U2AF2** ]   [ **U2AF1** ]  65 is critical for U1 recruitment, and we discuss the role of its RNA RNA annealing activity in this novel function of U2AF  [ **U2AF2** ]   [ **U2AF1** ]  65.  Ref: 12558503 Biochem J, 2003
- It inserts a conserved tryptophan into a hydrophobic pocket between helices A and B in a way that strikingly resembles part of the molecular interface in the U2AF  [ **U2AF2** ]   [ **U2AF1** ]  heterodimer.  Ref: 12718882 Mol Cell, 2003
- Structural Basis for the Molecular Recognition between Human Splicing Factors U2AF  [ **U2AF2** ]   [ **U2AF1** ]  65 and SF1 mBBP.  Ref: 12718882 Mol Cell, 2003
- The structure of the C terminal RRM RRM3 of human U2AF  [ **U2AF2** ]   [ **U2AF1** ]  65 complexed to an N terminal peptide of SF1 reveals an extended negatively charged helix A and an additional helix C.  Ref: 12718882 Mol Cell, 2003
- Recombinant U2AF35  [ **U2AF1** ]  RRM protein free and in complex with three different regions of U2AF65  [ **U2AF2** ]  was characterized by nuclear magnetic resonance spectroscopy.  Ref: 12297299 FEBS Lett, 2002
- It also appears that some RNA processing factors are required for viability e.g., U2AF  [ **U2AF2** ]   [ **U2AF1** ] , PUF60, SRp54, SAP49, PRP8, U1 70K.  Ref: 12810918 RNA< MedlineTA>RNA, 2002
- A novel function for the U2AF  [ **U2AF2** ]   [ **U2AF1** ]  65 splicing factor in promoting pre mRNA 3 end processing.  Ref: 12189174 EMBO Rep, 2002
- The S.pombe SF1 BBP, U2AF  [ **U2AF2** ]   [ **U2AF1** ]  59 and U2AF  [ **U2AF2** ]   [ **U2AF1** ]  23 are tightly associated in a novel complex that is required for complex A formation.  Ref: 12374752 EMBO J, 2002
- This pre formed SF1 U2AF  [ **U2AF2** ]   [ **U2AF1** ]  59 U2AF  [ **U2AF2** ]   [ **U2AF1** ]  23 complex may represent a streamlined mechanism for recognition of the branch site, pyrimidine tract and 3 splice site at the 3 end of introns.  Ref: 12374752 EMBO J, 2002
- Pre spliceosome formation in S.pombe requires a stable complex of SF1 U2AF  [ **U2AF2** ]   [ **U2AF1** ]  59 U2AF  [ **U2AF2** ]   [ **U2AF1** ]  23.  Ref: 12374752 EMBO J, 2002
- The structural basis for recognition between human splicing factors U2AF  [ **U2AF2** ]   [ **U2AF1** ]  65 and SF1 mBBP has recently been elucidated, setting the scene for understanding a key step in pre mRNA splicing and revealing a new conserved function for RRM proteins.  Ref: 12737811 Structure Camb , 2003
- The RRM3 of U2AF  [ **U2AF2** ]   [ **U2AF1** ]  65 shows no cross linking to the Py tract.  Ref: 12554879 RNA, 2003
- The essential splicing factors SF1 and U2AF  [ **U2AF2** ]   [ **U2AF1** ]  play an important role in the recognition of the pre mRNA 3 splice site during early spliceosome assembly.  Ref: 12718882 Mol Cell, 2003
- Therefore, we propose that U2AF  [ **U2AF2** ]   [ **U2AF1** ]  65 promotes 3 end processing, which contributes to 3 terminal exon definition.  Ref: 12189174 EMBO Rep, 2002
- Naturally occurring beta thalassaemia causing mutations within the pyrimidine tract reduces both U2AF  [ **U2AF2** ]   [ **U2AF1** ]  65 binding and 3 end cleavage efficiency.  Ref: 12189174 EMBO Rep, 2002
- Moreover, because U2AF  [ **U2AF2** ]   [ **U2AF1** ]  and the U1 snRNP are only associated transiently with the pre mRNA during the course of spliceosome assembly, our studies are difficult to reconcile with the current model that proposes that the SEX LETHAL blocks splicing at the second catalytic step, and instead argue that the SEX LETHAL protein acts after splice site recognition, but before catalysis begins.  Ref: 12490553 Development, 2003
- The requirements for S.pombe complex A formation pre mRNA sequence elements, protein splicing factors, SF1 BBP and both subunits of U2AF  [ **U2AF2** ]   [ **U2AF1** ]  are analogous to those of mammalian complex A.  Ref: 12374752 EMBO J, 2002
- Induced folding of the U2AF35  [ **U2AF1** ]  RRM upon binding to U2AF65  [ **U2AF2** ] .  Ref: 12297299 FEBS Lett, 2002
- The human essential splicing factor U2AF  [ **U2AF2** ]   [ **U2AF1** ]  U2 auxiliary factor consists of 35 and 65 kDa subunits which form a highly stable heterodimer in solution.  Ref: 12297299 FEBS Lett, 2002
- Both RRM1 and RRM2 of U2AF  [ **U2AF2** ]   [ **U2AF1** ]  65 and SXL can be cross linked to certain residues, with RRM2 showing a surprisingly high number of residues cross linked.  Ref: 12554879 RNA, 2003
- Over expression of either TcSR or the human SR protein associated splicing factor splicing factor 2 ASF SF2 in wild type Schizosaccharomyces pombe, provoked an elongated phenotype similar to that of fission yeast over expressing the SR containing splicing factor Prp2, a U2AF  [ **U2AF2** ]   [ **U2AF1** ]  65 orthologue.  Ref: 12615334 Mol Biochem Parasitol, 2003
- Significantly, a fusion protein containing U2AF  [ **U2AF2** ]   [ **U2AF1** ]  65, when tethered upstream of a cleavage polyadenylation site, increases 3 end cleavage efficiency in vitro and in vivo.  Ref: 12189174 EMBO Rep, 2002
- Similar genetic and biochemical approaches also link SEX LETHAL with the heterodimeric splicing factor, U2AF  [ **U2AF2** ]   [ **U2AF1** ] .  Ref: 12490553 Development, 2003
- We report that U2AF  [ **U2AF2** ]   [ **U2AF1** ]  65 can also promote the recruitment of U1 snRNP to weak 5 splice sites that are followed by uridine rich sequences.  Ref: 12558503 Biochem J, 2003
- Sex lethal and U2 small nuclear ribonucleoprotein auxiliary factor  [ **U2AF2** ]   [ **U2AF1** ]  U2AF65  [ **U2AF2** ]  recognize polypyrimidine tracts using multiple modes of binding.  Ref: 12554879 RNA, 2003
- The Drosophila splicing repressor Sex lethal SXL, which has two RRMs, can directly compete with the essential splicing factor U2AF  [ **U2AF2** ]   [ **U2AF1** ]  65, which has three RRMs, for binding to specific Py tracts.  Ref: 12554879 RNA, 2003
